# Friedreich Ataxia and nephrotic syndrome: a series of two patients

**DOI:** 10.1186/s12883-016-0526-2

**Published:** 2016-01-12

**Authors:** Julianna E. Shinnick, Charles J. Isaacs, Sharon Vivaldi, Kimberly Schadt, David R. Lynch

**Affiliations:** Departments of Pediatrics and Neurology, Children’s Hospital of Philadelphia, 502 Abramson Research Center, 3615 Civic Center Blvd, Philadelphia, PA 19104-4318 USA; Department of Physical Therapy, Department of Rehabilitation, Children’s Hospital of Philadelphia, King of Prussia Specialty Care and Surgery Center, 550 South Goddard Blvd, King of Prussia, PA 19406 USA; Perelman School of Medicine, University of Pennsylvania, Philadelphia, PA 19104 USA

**Keywords:** Friedreich Ataxia, Nephrotic Syndrome, Steroid

## Abstract

**Background:**

Friedreich Ataxia (FRDA) is a neurodegenerative disorder characterized by gait and balance abnormalities, sensory loss, weakness, loss of reflexes, and ataxia. Previously, two cases of FRDA and Nephrotic Syndrome (NS) have been reported. Here we report two additional individuals with NS and FRDA, providing further evidence for a possible connection between the two diseases and focusing on the neuromuscular responsiveness of one individual to corticosteroid treatment, an effect not previously described in FRDA.

**Case presentations:**

We describe two patients with FRDA also presenting with NS. The first patient was diagnosed with FRDA at age 5 and NS at age 7 following the development of periorbital edema, abdominal swelling, problems with urination, and weight gain. The second patient was diagnosed with NS at age 2 after presenting with periorbital edema, lethargy, and abdominal swelling. He was diagnosed with FRDA at age 10. Nephrotic syndrome was confirmed by laboratory testing in both cases and both individuals were treated with corticosteroids.

**Conclusions:**

Nephrotic syndrome may occur in individuals with FRDA, but was not associated with myoclonic epilepsy in our patients as previously described. It is unlikely that this association is coincidental given the rarity of both conditions and the association of NS with mitochondrial disease in model systems, though coincidental coexistence is possible. One patient showed neurological improvement following steroid treatment. Although neurological improvement could be attributed to the treatment of NS, we also identified some degree of steroid responsiveness in a series of patients with FRDA but without NS.

## Background

Friedreich Ataxia (FRDA) is a neurological disease resulting in gait and balance abnormalities, sensory loss, weakness, loss of reflexes, and ataxia. A recessive disorder, FRDA can also result in scoliosis, urinary dysfunction, diabetes mellitus, optic atrophy, hearing loss, sleep apnea, and hypertrophic cardiomyopathy [1–8]. The disease is caused by expanded guanine-adenine-adenine (GAA) repeats on both alleles of the FXN gene (*FXN*) in 98 % of patients. The remaining 2 % of individuals with FRDA have an expanded triplet repeat on one allele and a point mutation or deletion on the other. FRDA is a mitochondrial disease, as the deficient protein in FRDA (frataxin) is crucial for mitochondrial iron-sulfur cluster containing enzymes involved in oxidative phosphorylation and the Krebs cycle [9–12]. There is currently no therapy for FRDA, though several clinical trials are ongoing [13, 14].

Although few manifestations outside of the typical features have been identified in FRDA, one previous report suggests an association of nephrotic syndrome (NS) with FRDA [15]. In addition, related ataxias with coenzyme Q deficiency are associated with NS as are other mitochondrial disorders [16, 17]. Here we report 2 more individuals with NS and FRDA, providing further evidence for a possible connection between the two diseases and focusing on the responsiveness of one individual to corticosteroid treatment, an effect not previously described in FRDA.

### Case Presentations

Patient 1: The patient is a 13 year old female of European descent (GAA repeat lengths = 650, 1000). She presented with gait and balance difficulties at age 4 and was diagnosed with FRDA at age 5. At age 7, she developed periorbital edema, abdominal swelling, problems with urination, and a weight gain of 10 lbs over 9 months. She was diagnosed with idiopathic NS after laboratory testing revealing a urine protein of 2854 mg/dl and albumin of 2.2 g/dL. Though renal biopsy was not performed, specific causes of secondary nephrotic syndrome were ruled out by clinical criteria and serological testing. She received prednisone pulse therapy at 30 mg daily, which was tapered after 4 weeks. Her NS responded rapidly and urine protein levels normalized. She had 5 relapses of NS over the next 5 years, characterized by urine protein levels >100 mg/dL, all treated with 30–60 mg daily of prednisone pulse therapy. Clinical manifestations and laboratory parameters (proteinuria) of NS resolved following steroid treatment; surprisingly, neurologic improvements were also noted by her caregiver and physical therapist. Specifically, after her first treatment with prednisolone, the patient’s physical therapist noted that she had maintained range of motion in her heel cords and hamstrings and retained her skill level in balance as related to single leg stance over the course of a year. In the year following a second steroid treatment, the patient demonstrated decreased balance, coordination and range of motion. Subjectively her sense of fatigue decreased and her endurance improved. Objectively, her gait improved with a narrower base and fewer falls.

The effect was most prominent in a worsening of her neurologic abilities following scoliosis surgery. Following surgery she again developed NS and became unable to walk. Corticosteroid treatment led to recovery of renal function as well as ambulation. However, as her steroids were tapered, she lost ambulatory ability. This improved with another pulse of steroid treatment in the absence of NS.

Patient 2: The patient is a 25 year old male of Indian descent with FRDA (GAA repeats lengths = 650, 850). At age 2, the patient presented with periorbital edema, lethargy, and abdominal swelling. Idiopathic NS was confirmed following urine protein testing. He developed gait difficulties at age 9 and was diagnosed with FRDA at age 10. He had been treated with chronic steroids from age 2 to age 10, with doses ranging from 15 mg every other day to 60 mg per day during episodic flares. In response to the onset of gait problems, the patient’s nephrologist switched him from steroid treatment to 100 mg of cyclophosphamide for 3 months. He has not had any relapses of NS since then. However, the patient’s ataxia worsened after the discontinuation of steroids.

Given the improvement of the first subject on corticosteroid treatment, we examined the records of the Children’s Hospital of Philadelphia (CHOP) and the Collaborative Clinical Research Network (CCRN) for other individuals reporting responses to corticosteroids prescribed for other indications in a retrospective review (Table 1) [18]. Nine people with FRDA experienced improved balance, gait or speech with corticosteroid or other immunomodulatory therapy, and no individuals were identified with significant steroid dependent worsening. One other patient treated with steroids showed improvement, although it is unclear whether improvement was due to corticosteroids or other medications initiated at the same time. All patients besides patients 1 and 2 described had normal kidney function.

**Table 1:** Patients with FRDA treated with steroids

Patient No. GAA repeats	Age of FRDA onset	Clinical course	Phenotype^a^	Immunomodulator	Dose	Duration	Reason for steroid treatment	Response	Age of steroid treatment
Patient 1 described 650, 1000	4	Began using wheelchair at age 10; scoliosis surgery at age 13	Ataxia, loss of balance, loss of sensation, leg cramps, tremors, hypertrophic cardiomyopathy, scoliosis, fatigue	Prednisolone, oral	30 BID-50 QD	26 months over 6 years	Nephrotic syndrome	Recurrent neurologic improvement coincident with steroid dosing	8–14
Patient 2 described 650, 950	10	Began using wheelchair at age 12	Ataxia, loss of balance, loss of sensation, spasms, hypertrophic cardiomyopathy, arrhythmia, scoliosis	Prednisone, oral	unknown	8 years	Nephrotic syndrome	Possible delay in presentation	2–10
Patient 3 500, 570	15	Began using cane at 23, wheelchair at 29	Ataxia, loss of balance, loss of sensation, leg spasms, restless legs, scoliosis, sleep apnea	Prednisone and Medrol, oral	8 QAM	4–5 months	Chronic inflammatory demyelinating polyneuropathy (likely misdiagnosis)	Mild improvements in gait, eventual progression	18
Patient 4 725, presumed point mutation	7	Began using wheelchair at 17	Ataxia, loss of balance, loss of sensation, leg spasms, restless legs, sleep apnea, fatigue, hypertrophic cardiomyopathy, scoliosis	Prednisone, oral	15–30 mg BID	7 months	Chronic inflammatory demyelinating polyneuropathy (likely misdiagnosis)	Improvements in balance, eventual progression	12
Patient 5 unknown	unknown	Unknown	Unknown	Prednisone	unknown	unknown	Rib fractures	Improvement in Gait	unknown
Patient 6 1000, 1000	3	Began using wheelchair at age 7	Ataxia, loss of balance, loss of sensation, leg spasms, restless legs, increased tone, tremor, hypertrophic cardiomyopathy, scoliosis	Prednisolone	unknown	unknown	Chronic inflammatory demyelinating polyneuropathy (likely misdiagnosis)	Improvement in gait and strength, eventual progression	3
Patient 7 1113, point mutation	2	Began using walker at age 5	Ataxia, loss of balance, loss of sensation, leg spasms, sleep apnea, hypertrophic cardiomyopathy, scoliosis	Methylprednisolone, pulse therapy	30 mg QD	5 days	Pneumonitis	No change in gait, balance, improved energy	6
Patient 8 766, 1000	7	Able to walk without assistive device	Minimal ataxia, loss of balance, no scoliosis or cardiac screening	Solumedrol, oral	5 mg TID	5 months	Unclear	Sustained improvements in balance, eventual progression	9
Patient 9 41, 696	43	Began using a walker at age 58	Ataxia, loss of balance, loss of sensation, leg spasms, increased tone	Depomedrone, injection	80 mg QD	7–9 years	Sciatic pain	Improvements in gait and speech	49
Patient 10 966, 1099	2	Able to walk without assistive device	Ataxia, loss of balance, loss of sensation, fatigue	Prednisolone, oral	30 mg OPD	3 days	Acute laryngotra-cheitis	Improvements in falling and fatigue	8

^a^Phenotype at closest exam to steroid use

There are several limitations to this study. Improvement in the context of corticosteroid treatment often occurred in the context of significant clinical changes, such as NS in the case of the two cases presented and surgery or trauma in other FRDA patients treated with steroids. Furthermore, in two cases, steroid treatment was initiated following intravenous immunoglobulin G treatment prescribed for chronic inflammatory demyelinating polyneuropathy and Guillain-Barre syndrome, both likely misdiagnoses for Friedreich Ataxia. This raises the possibility that in these cases concomitant intravenous immunoglobulin G treatment was at least partially responsible for noted improvements.

## Conclusions

We describe two patients with FRDA and NS, one of whom demonstrated significant improvement with corticosteroid treatment. An association between FRDA and NS was initially reported in a single family in association with myoclonic epilepsy. Epilepsy was not identified in our subject, thus dissociating such findings (as predicted previously). As NS and FRDA are both rare, the present association in multiple unrelated subjects is unlikely to be coincidental. Furthermore, nephrotic syndrome has been documented in a variety of mitochondrial cytopathies and mutations in the synthesis of Coenzyme Q_10_ cause a subset of steroid-resistant nephrotic syndrome cases [19, 20]. Mitochondrial dysfunction is central to the pathophysiology of Friedreich Ataxia [21]. Lack of frataxin disrupts the production of iron-sulfur clusters and increases levels of intracellular ROS in animal models,patient biopsies and FRDA fibroblasts, suggesting increased oxidative stress in FRDA cells [10, 22–24]. Although the typical symptomatology of FRDA results from mitochondrial dysfunction in the spine, cerebellum and heart, we hypothesize that the cases of nephrotic syndrome in FRDA described result from mitochondrial involvement in renal cells.

Interestingly one of our subjects showed substantial neurological improvement following steroid treatment. No improvement in the non-neurological symptoms of FRDA were noted. Although this might represent a secondary event associated with improvement in her NS, we identified some degree of steroid responsiveness in her independent of NS and in other patients with FRDA. This could represent an anti-inflammatory effect (as two other patients responded transiently to intravenous immunoglobulin G) or could be the result of other effects of corticosteroids such as increased strength as a compensatory mechanism for balance dysfunction. The former possibility seems most likely, as a secondary inflammatory response in FRDA has been revealed in autopsy studies and in alterations of immune pathways in microarray analysis [11, 12]. This is felt to be the mechanism behind the well-documented response of Duchenne muscular dystrophy to corticosteroids [25, 26].

On the molecular level, it is possible that steroids modify the oxidative stress caused by frataxin deficiency and subsequent mitochondrial disease. This change in oxidative stress was hypothesized to be a cause for neurological improvement following steroid treatment in ataxia-telangiectasia [27]. The catabolic effect of steroids could provide a mechanism for the improvements seen in FRDA, as altered lipid metabolism has been documented in rat myocytes with diminished frataxin levels and in *Drosophilia melanogaster* [28, 29]. The present patient, taken with previous basic and scientific research, suggests the importance of pilot studies examining the efficacy of pulse steroid treatment as a potential therapy in FRDA.

### Consent

Written informed consent was obtained from the patients for review of their records for publications. A copy of the written consent is available for review.

## Abbreviations

FRDA: Friedreich’s Ataxia
GAA: Guanine-Adenine-Adenine
NS: Nephrotic Syndrome
CHOP: Children’s Hospital of Philadelphia
CCRN: Collaborative Clinical Research Network
